# Effect of Organic Inputs and Solarization for the Suppression of *Rhizoctonia solani* in Woody Ornamental Plant Production

**DOI:** 10.3390/plants8050138

**Published:** 2019-05-24

**Authors:** Fulya Baysal-Gurel, Md Niamul Kabir, Prabha Liyanapathiranage

**Affiliations:** Department of Agricultural and Environmental Sciences, Otis L. Floyd Nursery Research Center, Tennessee State University, McMinnville, TN 37110, USA; mkabir@my.tnstate.edu (M.N.K.); pliyanap@my.tnstate.edu (P.L.)

**Keywords:** biofumigation, solarization, compost, mustard meal, Rhizoctonia root rot, flowering cherry

## Abstract

Soilborne diseases are the most economically significant problem faced by Southern region nursery producers. The goal of this research was to improve Rhizoctonia root rot disease management through the use of soil solarization alone and in combination with biofumigant cover crops—arugula ‘Astro’ (*Eruca vesicaria* ssp. *sativa*), mustard green ‘Amara’ (*Brassica carinata*), and turnip ‘Purple top forage’ (*B. rapa*); good quality compost and mustard meal amendment. The experiments were established as on-farm trials in 2016 and 2017 with prevalent *Rhizoctonia solani* population in propagation beds. All three biofumigant cover crops, arugula ‘Astro’, mustard green ‘Amara’, and turnip ‘Purple top forage’ in combination with solarization were able to reduce the Rhizoctonia root rot in flowering cherry ‘Kwanzan’ plants in nursery propagation beds. Compost amendment increased the flowering cherry rooted cuttings growth (plant weight, root weight, and plant height) compared to other treatments. Soil solarization in combination with cover crops and organic inputs could be used as part of an integrated approach to manage Rhizoctonia root rot in nursery crop propagation beds.

## 1. Introduction

Soilborne pathogens can survive in the soil as resistant propagules, such as chlamydospores, sclerotia, thick-walled conidia, or hyphae, or survive in plant roots and crop residues [1]. When conditions are favorable, soilborne pathogens (*Rhizoctonia* spp., *Phytophthora* spp., *Sclerotinia* spp., *Pythium* spp., *Verticillium* spp., and *Fusarium* spp.) can cause severe damage to a large variety of crops including woody ornamentals, particularly in propagation systems. Most of the time, compared to mature plants, young seedlings/liners are more susceptible to soilborne diseases [2]. Crop losses of flowering cherry ‘Kwanzan’ rooted cuttings in propagation beds can exceed 60% due to Rhizoctonia root rot [3]. Soilborne diseases have been conventionally controlled by performing soil fumigation using methyl bromide [4] or metam sodium [5,6]. Cover crops in the family Brassicaceae also have the ability to suppress soilborne pathogens through the hydrolysis of glucosinolates (GSL) into isothiocyanate (ITC), a natural biofumigant [7,8]. Plants that contain GSL have long been known for their specific flavor and smell, which is related to the presence of these compounds [9]. So far, more than 20 aromatic and aliphatic isothiocyanates and potential allelochemicals have been identified from *Brassica juncea*, *B. napus*, *B. campestris*, and *B. nigra* [10,11]. Depending on the GSL profile, each *Brassica* species may release different cyanates. Fungi are inhibited by pure isothiocyanates as was first described by Walker and his colleagues in 1937 [12]. Mycelial growth inhibition of *Rhizoctonia solani*, *Pythium irregular,* and *Fusarium graminearum* by ITCs has been identified previously [13]. *Brassica* plant species and cultivars have varying concentrations and chemical compositions of GSL compounds in their tissues which may affect the biofumigation efficacy [14]. The AITC (allyl isothiocyanate) compound released from chopped *B. juncea* has strong microbial activity against *R. solani* [15]. Also, arugula (*Eruca vesicaria* ssp. *sativa*) and sunn hemp (*Crotalaria juncea*) have the ability to inhibit the mycelial growth of *R. solani* possibly due to the different chemical types found in these plants [16,17]. 

Soil solarization is known as hydrothermal process to increase soil temperature under the transparent plastic to create unfavorable conditions for weeds, insects, and soilborne plant pathogens [18,19]. The soil solarization process is considered one of the novel approaches because it can only reduce mesophyllic organisms (which includes most of the pests and soilborne plant pathogens) without killing various beneficial microorganisms (growth-promoting *Bacillus* spp. and mycorrhizal fungi) [20,21]. Solarization with increased heat can also directly weaken and inactivate different soilborne plant pathogens, and make them defenseless against different microorganisms, to various soil fumigants or even to atmospheric changes in the solarized soil [20,22,23]. Katan and his colleagues reported a long-term effect of soil solarization against *F. oxysporum* f. sp. *vasinfectum* [24]. A number of *Phytophthora* species have been reported as sensitive to high temperatures obtained from solarized soils [25,26]. In South Africa, it has been reported that six weeks of solarization can completely eliminate *P. cinnamomi* [27] and in Australia, solarization has been used to control the impact of *P. cambivora* in cherry production [28]. It has been documented that solarization reduced the populations of the following soilborne pathogens including *Verticillium dahliae* [18,29,30,31,32], *Fusarium* spp. [18,31,33,34], *P. ultimum* [18,32,35,36,37,38,39], *Rhizoctonia* sp. [18,32,40,41,42], *Agrobacterium tumefaciens* [43], *Phytophthora* spp. [18,25,26,30,33,44,45], *Sclerotium rolfsii, P. aphanidermatum,* and *Plasmodiophora brassicae* [18,33]. Solarization effectiveness is dependent on the following factors such as intensity of sunlight, air temperature, soil moisture, length of day, soil color, and structure [18,21,39,46,47]. In regions like the southeastern part of the United States where rain showers and high summer temperatures are prevalent, solarization can be less effective as the rain cools down the temperature and also reduces solar radiation under the plastic [33]. 

When considering the published literature, soil solarization alone may not always be effective in controlling soilborne pathogens but biofumigation combined with solarization was found to be more effective in controlling soilborne pathogens, nematodes, and weeds [19,48]. In several cases adding soil amendments increased the performance of solarization [34,49,50]. It is recommended to use cruciferous residues which contain degradation products of GSL, as soil amendments in combination with the solarization process [15]. It has been reported that the use of cabbage leaf debris with the soil solarization process can increase the efficiency to control root diseases and suppress several soilborne pathogens [18,34,50,51]. Incorporation of *Brassica* has also been found to result in an increase in soil organic matter, mineral nutrients, and effect the colonization of arbuscular mycorrhizal fungi [52]. Not only can green manures provide an alternate approach for controlling soilborne diseases, they can also increase the availability of essential nutrients and stimulate the populations of beneficial microorganisms in the soil [53]. Adding organic inputs into the soil can increase the effectiveness of solarization [34]. Therefore, more studies are necessary to evaluate the combined effect of biofumigant cover crops and soil solarization against different soilborne pathogens.

This study focused on cultural approaches including soil solarization alone and in combination with biofumigant cover crop incorporation (arugula ‘Astro’ (*E. vesicaria* ssp. *sativa*), mustard green ‘Amara’ (*B. carinata*), and turnip ‘Purple top forage’ (*B. rapa*) (solarization and cover crop), and also compost cow manure and mustard meal amendment to evaluate their effectiveness in controlling soilborne pathogens prevailing at on-farm nursery crop propagation beds.

## 2. Results

Rhizoctonia root rot severity was high in the 2016 on-farm experiment; the mean root rot severity was 67.40% in the non-treated control flowering cherry ‘Kwanzan’ plants (Table 1). All the tested cover crops—arugula ‘Astro’, mustard green ‘Amara’, and turnip ‘Purple top forage’ in combination with solarization significantly reduced Rhizoctonia root rot severity on flowering cherry plants compared to the non-treated control plants and reduced Rhizoctonia root rot severity compared to the solarization alone, compost, and mustard meal amendments (*P* = 0.0012). Compost amendment significantly increased plant weight compared to all the tested cover crops in combination with solarization and non-treated control, and increased plant weight compared to mustard meal amendment and solarization alone (*P* = 0.0090). Compost amendment also significantly increased root weight compared to all the tested cover crops in combination with solarization, mustard meal amendment, and non-treated control and increased root weight compared to solarization alone (*P* = 0.0010). Compost amendment significantly increased plant height compared to the non-treated control, solarization alone, and turnip ‘Purple top forage’ cover crop in combination with solarization and increased plant height compared to mustard meal amendment, arugula ‘Astro’, and mustard green ‘Amara’ cover crops in combination with solarization (*P* = 0.0198) (Table 1). 

In the 2017 on-farm experiment, Rhizoctonia root rot disease severity in ‘Kwanzan’ flowering cherry was higher compared to 2016 with non-treated control plants showing 72.47% disease severity. All of the treatments significantly reduced Rhizoctonia root rot disease severity in flowering cherry ‘Kwanzan’ plants compared to the non-treated control (*P* < 0.0001). There were no significant differences between the treatments in Rhizoctonia root rot severity. Also, there were no significant differences between the treatments in plant weight (*P* = 0.0698), root weight (*P* = 0.0685), and plant height (*P* = 0.4856) (Table 2). 

**Figure 1:**
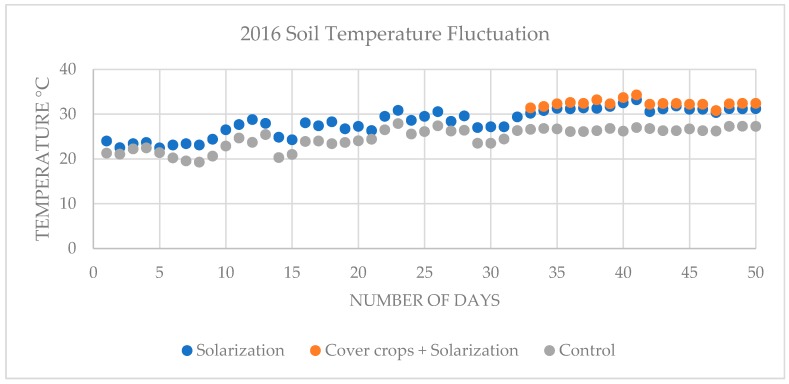
Soil temperature fluctuation in the top 15 cm of solarized beds, cover crops incorporated solarized beds and control non-treated, non-solarized beds in the 2016 on-farm experiment.

**Figure 2:**
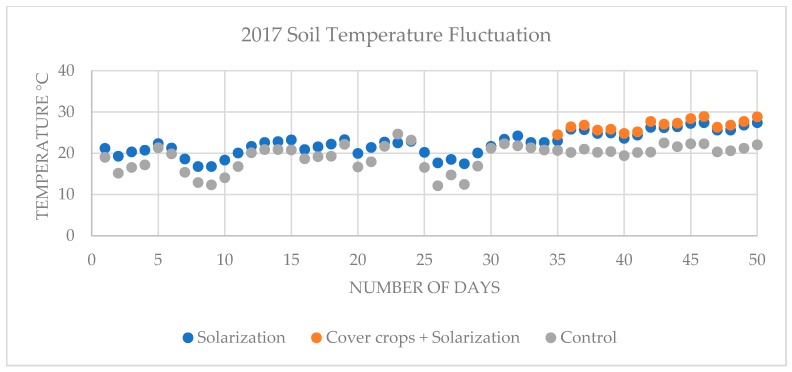
Soil temperature fluctuation in the top 15 cm of solarized beds, cover crops incorporated solarized beds and control non-treated, non-solarized beds in the 2017 on-farm experiment.

In the 2016 on-farm experiment, average soil temperature of solarized beds was 28.46 °C between June 20 and August 8 and 31.29 °C between July 25 and August 8; average soil temperature of cover crops incorporated solarized beds was 32.38 °C between July 25 and August 8; average soil temperature of control non-treated, non-solarized beds was 24.81 °C between June 20 and August 8 and 26.61 °C between July 25 and August 8 (Figure 1). In the 2017 on-farm experiment, average soil temperature of solarized beds was 22.63 °C between June 19 and August 7 and 25.69 °C between July 24 and August 7; average soil temperature of cover crops incorporated solarized beds was 26.75 °C between July 24 and August 7; average soil temperature of control non-treated, non-solarized beds was 19.43 °C June 19- August 7 and 20.95 °C between July 24 and August 7 (Figure 2).

In both the 2016 and 2017 on-farm experiments, highest root rot plant pathogen recovery was observed in compost amended and non-treated control plants (Table 3). In the 2016 experiment, flowering cherry ‘Kwanzan’ plants grown in mustard green ‘Amara’ or arugula ‘Astro’ incorporated plots in combination with solarization had a smaller percent recovery of *R. solani* from root samples compared to the non-treated control plants. There were no differences between turnip ‘Purple top forage’ incorporated plots in combination with solarization, solarization alone, compost, mustard meal amendment, and non-treated control plots in percent recovery of *R. solani* from root samples. In the 2017 experiment, plants grown in turnip ‘Purple top forage’, mustard green ‘Amara’, or arugula ‘Astro’ incorporated plots in combination with solarization and solarization alone plots had a lower percent recovery of *R. solani* from root samples compared to the non-treated control plants. There were no differences between compost, mustard meal amended plots, and non-treated control plots in percent recovery of *R. solani* from root samples.

## 3. Discussion

Fumigants have been widely used in agriculture to control soilborne diseases and obtain a higher crop yield. Methyl bromide and chloropicrin are the most commonly used fumigants to control fungal pathogens [16]. Metham sodium is also one of the fumigants used to control soilborne pathogens [54] but metham sodium is decreasing microbial biomass and microbial activity and affecting the microbial community structure [55]. Intensive crop farming systems with short crop rotations used in modern agriculture has increased the soilborne pathogen inoculum densities [56]. Even though nursery producers heavily rely on conventional fungicides, fungicide resistance continues to generate soilborne disease control problems in woody ornamental crops. Because of nursery crop losses due to resistance, as well as environmental and health concerns, it is critical to identify alternative, environmentally friendly approaches to control soilborne diseases in order to meet the needs of the nursery industry for healthy and high-quality plants. 

Biofumigation is the incorporation of plant material into the soil as a green manure containing GSL compounds to suppress soilborne pathogens [57]. To perform biofumigation, GSL containing crops need to be planted in a rotation and when the plants reach the flowering stage (which has higher concentration of GSL in the tissues) they need to be chopped and incorporated into the soil at a depth of 10–20 cm [9]. When the plant materials are chopped, GSL hydrolyzed products isothiocyanates (ITC) are released [58]. Biofumigation can also contribute to improved soil texture, soil nutrient content, and water infiltration [59]. Amending soil with plant residues can improve microbial community composition and increase the competition or antagonism among soil microbes, thereby decreasing soilborne pathogen activity [60,61]. In this study, all three biofumigant cover crops—arugula ‘Astro’ (*Eruca vesicaria* ssp. *sativa)*, mustard green ‘Amara’ (*Brassica carinata*), and turnip ‘Purple top forage’ (*B. rapa*)—in combination with solarization were able to significantly reduce the Rhizoctonia root rot in flowering cherry ‘Kwanzan’ plants in nursery propagation beds. *Brassica* plant species and cultivars have varying concentrations and chemical compositions of GSL compounds in their plant tissues and those may affect biofumigation efficacy [14]. In a previous study, the allyl ITC compound released from chopped *B. juncea* had strong microbial activity against *R. solani* [15]. Also, arugula (*E. vesicaria* ssp. *sativa*) and sunn hemp (*C. juncea*) had the ability to inhibit the mycelia growth of *R. solani* possibly due to the different chemical types found in these plants [16,17]. Larkin and Griffin [54] have reported that strawberry plots biofumigated with *B. juncea* reduced *R. solani* inoculum levels by 40–56% and also reduced the severity and incidence of root disease symptoms. In this study, plots biofumigated with cover crops (*E. vesicaria* ssp. *sativa, B. carinata,* and *B. rapa)* in combination with solarization had 30–35% less Rhizoctonia root rot disease in flowering cherry plants compared to the non-treated plots. 

Soil solarization procedure is considered as a process of pasteurization depending on the accumulation of solar energy which heat the soil and kill soilborne pathogens. In the southeastern part of the United States, solarization has been used to control *R*. *solani, Fusarium* spp., *P. nicotianae,,* and *Didymella bryoniae* [33,41,50,62]. Efficient pasteurization could occur when soil maintains 42–52 °C temperatures in the top 15 cm, which are lethal to soilborne pathogens reported to infest plants in arid climates [18,46,63]. On the other hand, management of soilborne pathogens by solarization is less effective in the regions where climate interrupts the heat trapping process of the soil [33,41,64,65]. Site to site temperatures could vary depending on soil/propagation substrate color, soil/propagation substrate moisture, and amount of clay, sand, and silt content into the soil/propagation substrate. All of these factors could play an important role in the accumulation of solar temperature into the soil. In this study, soil temperature changes (~5.6 °C) were observed in the top 15 cm of all plots between 2016 and 2017 on-farm experiments. However, we did observe ~1.1 °C increase in the soil temperature of cover crops incorporated plots in combination with solarization compared to solarization alone for both 2016 and 2017 experiments. This result is consistent with previous studies [36,49,66,67] which also observed that the combined utilization of biofumigation or organic amendments such as incorporated residue of cover crop or animal manure with soil solarization techniques increased soil temperature by 1–3 °C. 

Phytotoxicity is one of the important factors that need to be considered when selecting cover crops to perform biofumigation. Although biofumigation can suppress soilborne pathogens, if the incorporated plant material negatively affects growth of the fallowing crop due to phytotoxicity, it will not be considered as a tool in disease management. During our experiments we did not observe any phytotoxicity in flowering cherry ‘Kwanzan’ plants with any of the tested cover crops. 

Compost and mustard meal amendments have also been shown to reduce Rhizoctonia root rot severity in the propagation beds, although the effects have been generally smaller and more variable compared to other treatments. The use of organic amendments such as animal manure, green manure, and seed meals has been proposed to improve soil structure and fertility and decrease the incidence of diseases caused by soilborne pathogens [68]. In this study, higher Rhizoctonia root rot severity was not always directly associated with lower plant growth such as plant weight, root weight, and plant height during on-farm trial periods. However, the nursery producers hold the rooted cuttings for longer duration in their propagation beds and this may result an inverse correlation between disease severity and plant growth. We also observed that compost amendment increased the flowering cherry rooted cuttings growth (plant weight, root weight, and plant height) compared to other treatments most likely due to effects on chemical, physical, and biological properties. 

An integrated disease management approach incorporating cultural, chemical, and biological techniques should be used to control soilborne pathogens. The results of this study indicate that cover crops (arugula ‘Astro’ (*Eruca vesicaria* ssp. *sativa)*, mustard green ‘Amara’ (*Brassica carinata*), and turnip ‘Purple top forage’ (*B. rapa*)) can be used in combination with solarization to reduce Rhizoctonia root rot in nursery crop propagation beds. This study only examined cultural approaches, while applications of fungicides with different modes of action in a rotation program to support cultural approaches might provide enhanced *Rhizoctonia* control. Nursery producers could benefit from using solarization in a combination with cover crop as part of an integrated strategy to manage Rhizoctonia root rot.

## 4. Materials and Methods

### 4.1. Study Location, Pathogen Identification and Experimental Design

Baysal-Gurel Lab received flowering cherry (*Prunus serrulata* ‘Kwanzan’) rooted cutting samples with root sot symptoms from a commercial nursery in Warren Co., Tennessee, in 2015 and the problem was identified as Rhizoctonia root rot caused by *Rhizoctonia solani* Kühn (teleomorph = *Thanatephorus cucumeris* (A.B. Frank) Donk) using culturing, PCR, and subsequent DNA sequencing. During the same year, propagation substrate samples were also collected from the beds using a probe (Model J soil sampler-60 cm, Item No: 6505, Spectrum technologies, Aurora, IL, USA) for the identification of prevalent soilborne pathogens. Substrate collected from each propagation bed was thoroughly mixed by hand and maintained in the dark at 4˚C until analysis. Samples were cultured on different growth media such as PARPH-V8, water agar, and acidic potato dextrose agar (a-PDA) media with serial dilutions for the identification of soilborne pathogens. Pathogens were purified and morphologically characterized from the cultures and then identified by PCR; the ribosomal DNA internal transcribed spacer (ITS) region was amplified by PCR using the primer pair ITS1 and ITS4 [69] and was sent for subsequent DNA sequencing (Eurofins Genomics, Louisville, KY, USA). To complete Koch’s postulates, ‘Kwanzan’ cherry cuttings grown on propagation substrate (10 × 10 cm pot containing 1kg sterilized substrate) were inoculated with identified pathogens *R. solani, Fusarium solani*, *F. oxysporum*, and *Pythium rosratifingens* in a greenhouse (21−23 °C) and the severity of root rot was assessed visually, using a scale of 0−100% total root system affected after a period of time to allow disease development (45−60 days). Plants were drench-inoculated in the substrate with a mycelial slurry suspension of 5-day old cultures of each pathogen (1 petri dish/1 L of sterilized water and 100 mL/plant) grown separately on PDA. Control plants were drenched with a PDA slurry in the substrate and maintained in the same environment. Pathogenicity test was designed as complete randomized design with five replications. Only ‘Kwanzan’ cherry rooted cuttings inoculated with *R. solani* developed root rot symptoms and microscopic examination revealed the same pathogen morphology as the original isolate. *R. solani* was consistently re-isolated from symptomatic root tissues. All non-inoculated control plants remained symptom-free and *Rhizoctonia* was not isolated from the tissue. 

Field experiments were conducted at the same commercial nursery with a randomized complete block design with three replications in 2016 and 2017. Solarization alone, arugula ‘Astro’ (*Eruca vesicaria* ssp. *sativa)*, mustard green ‘Amara’ (*Brassica carinata*), and turnip ‘Purple top forage’ (*B. rapa*) as cover crops in combination with solarization, mustard meal, and cow manure compost were evaluated for their ability to control Rhizoctonia root rot on flowering cherry (*P. serrulata* ‘Kwanzan’) cuttings in propagation beds (Table 4). Cover crops were selected based on findings of previous bioassays [3]. The rates were based on the suppliers’ recommendation per acre.

### 4.2. Propagation Bed Preparation

Propagation bed preparations were started in November 2015 for the 2016 experiment and in November 2016 for the 2017 experiment. Each year, different beds were used for the experiments. Land was prepared by removing all weeds and plant debris. Propagation substrate contained 40% coarse sand and 60% ground pine bark. The experiment was conducted in pre-existing raised beds, where each bed was 11.68 × 1.12 m ~14 m^2^. Pelletized lime (Agri Perl, the pelletizing company, Gordonsville, TN, USA) at 4.54 kg was added into the bed and the soil was tilled (Honda FRC800 tiller) in March 2016 and March 2017. Granulated fertilizer 13-13-13 with trace elements (Super Rainbow^®^, Agrium U.S. Inc., Denver, CO, USA) at 5.54 g was added into the beds. Beds were irrigated using sprinklers and each sprinkler nozzle was 60 cm apart (18 sprinkler nozzles within the bed). Each bed was 1.50 × 1.12 m and in between each plot; ~20 cm untreated zone was prepared using a crowbar to demarcate the area per each plot and to prevent cross contamination. Propagation beds were demarcated using polythene strips.

### 4.3. Application of Organic Inputs and Solarization

Cover crop seeds were seeded with recommended rates into the propagation beds with 50 g of coarse sand for even distribution in the 2016 experiment on May 11, and for the 2017 experiment on May 31 (Table 1). Sprinklers were set up for irrigation daily as 15 sec/every 10 min from 9:30 a.m. to 5:30 p.m. When the cover crops reached to the flowering stage in late July in both experiments, plants were uprooted from each plot and chopped into small pieces with shears (particle size about 1 cm). Chopped plant materials were incorporated 15 cm deep into the same plots they were grown using ploughs. Cover crop incorporated beds were covered in the last week of July in both experiments using transparent polythene (clear poly polyethylene sheeting, 3.0-mil, Wrap Bros, Chicago, IL, USA) and the edges were sealed using sand. Mustard meal and cow manure compost were added into the assigned propagation beds in first week of August in both experiments according to the recommended rates (Table 1). After 14 days, polythene was removed from the biofumigated beds and all the beds were tilled using a tiller (Honda FRC800 tiller, American Honda Motor Co., Inc., Swepsonville, NC, USA). In the 2016 experiment, solarization alone-plots were covered with polythene on June 20 and uncovered on August 8. In the 2017 experiment, solarization alone-plots were covered with polythene on June 19 and uncovered on August 7. Before starting the solarization, the beds were tilled again and wetted to trap more temperature with hand held irrigation. Non-treated propagation beds served as controls. In the 2016 and 2017 on-farm experiments, soil temperature data of solarized beds, cover crops incorporated solarized beds, and control non-treated, non-solarized beds were collected using WatchDog (Spectrum Technologies, Inc., Aurora, IL, USA).

### 4.4. Soft Wood Cutting Preparation

Flowering cherry (*P. serrulata* ‘Kwanzan’) cuttings were taken from the mother plants at the same commercial nursery in Warren Co., McMinnville, TN, USA. Cuttings were 20–25 cm in height and three leaves were kept remaining. A slant cut was made at the end of the stem and from the remaining leaves; half of the leaves were cut and removed. Cuttings were dipped into 1% 3-indo-beuretic Acid (IBA) (Harmodin^®^ 3, OHP Inc, Mainland, PA, USA) and the cuttings were stacked with wet cloths until transplanting.

### 4.5. Transplanting and Aftercare

Ten flowering cherry cuttings were transplanted into each propagation bed. All beds were covered using black shade and wire mesh cloth. Sprinklers were used to water the plots 15 sec/10 min. Three weeks after transplanting, insecticides, Orthene^®^ 97 Soluble (AMVAC Chemical Corporation, Los Angeles, CA, USA) at 3 tsp/15 L and Malathion 57% (Control Solutions, Inc, Pasadena, TX, USA) at 1 tsp/3.8 L, were applied to all plots. Five weeks after planting, Orthene at 3 tsp/15 L and Sevin ready-to-use 5% dust (TechPac, LLC. Atlanta, GA, USA) were applied to control insects. By the sixth week, irrigation was cut down to 10 sec/10 min and by the eighth week, it was further cut back to 5 sec/10 min. After the sixth week, shade was partially removed the morning time for the hardening of the cuttings. From the ninth week, sprinklers were turned on between 12:00 pm and 5:30 pm only two days a week and by the tenth week, beds were watered only using hose for 3–4 days a week. Fertilizer Peters Professional^®^ 20-20-20 (ICL Specialty Fertilizers, Summerville, SC, USA) was applied by the tenth week. Fertilizer at 110 g rate was diluted in 22.7 L water and applied to all plots. Shade was completely removed in the tenth week and it was placed only if wilting was observed.

### 4.6. Crop Health Assessment

Rooted cuttings were removed from the beds/plots and bagged. Plant height, plant total fresh weight, and root fresh weight (roots were cut from the plant at the base of the root collar) were recorded after the trial. Additionally, the severity of Rhizoctonia root rot was assessed visually, using a scale of 0−100% total root system affected at the end of the trials. *Rhizoctonia* infection was determined by plating ten randomly selected root samples (~1 cm long) from the root tips of each plant on *Rhizoctonia* semi-selective medium [70]. Plates were incubated at 25 °C for 48 h in an incubator and the presence or absence of *Rhizoctonia* growth surrounding each root sample was recorded after morphological observation.

### 4.7. Data Analysis

Plant height, plant weight, root weight, disease severity, and percent recovery of *R. solani* from root samples were compared among treatments. Analysis of variance of all the data sets was performed using the general linear model’s procedure with SAS statistical software (SAS Institute Inc., Cary, NC, USA) and means were separated using Tukey test.

## Figures and Tables

**Table 1:** Mean Rhizoctonia root rot severity and plant growth data of flowering cherry plants treated with solarization alone or in combination with cover crops, compost, and mustard meal, 2016 on-farm experiment.

Treatment	Root Rot Severity (%) ^x^	Plant Weight (g)	Root Weight (g)	Plant Height (cm)
Arugula ‘Astro’ + Solarization	33.73 b^y^	18.23 b	7.00 b	21.52 ab
Mustard green ‘Amara’ + Solarization	34.38 b	18.60 b	7.32 b	22.68 ab
Turnip ‘Purple top forage’ + Solarization	38.77 b	18.83 b	6.43 b	21.00 b
Solarization alone	48.00 ab	22.00 ab	8.41 ab	19.93 b
Compost	49.67 ab	27.20 a	10.70 a	26.37 a
Mustard meal	49.41 ab	21.87 ab	7.30 b	21.72 ab
Non-treated control	67.40 a	17.62 b	5.39 b	20.76 b
*P*-value	0.0012	0.0090	0.0010	0.0198

^x^ Disease severity was based on percentage of roots affected. ^y^ Values are the means of three replicates; treatments followed by the same letter within a column are not significantly different at *P* ≤ 0.05.

**Table 2:** Mean Rhizoctonia root rot severity and plant growth data of flowering cherry plants treated with solarization alone or in combination with cover crops, compost, and mustard meal, 2017 on-farm experiment.

Treatment	Root Rot Severity (%)^x^	Plant Weight (g)	Root Weight (g)	Plant Height (cm)
Arugula ‘Astro’ + Solarization	41.48 b^y^	16.47 a	6.47 a	20.58 a
Mustard green ‘Amara’ + Solarization	44.18 b	17.98 a	7.91 a	20.98 a
Turnip ‘Purple top forage’ + Solarization	37.00 b	18.13 a	8.08 a	20.64 a
Solarization alone	47.79 b	16.41 a	6.64 a	20.67 a
Compost	37.10 b	18.97 a	7.90 a	21.74 a
Mustard meal	37.65 b	18.24 a	7.92 a	20.29 a
Non-treated control	72.47 a	17.80 a	7.48 a	20.27 a
*P*-value	<.0001	0.0698	0.0685	0.4856

^x^ Disease severity was based on percentage of roots affected. ^y^ Values are the means of three replicates; treatments followed by the same letter within a column are not significantly different at *P* ≤ 0.05.

**Table 3:** Percent recovery of *Rhizoctonia solani* from root samples treated with solarization alone or in combination with cover crops, compost and mustard meal.

Treatment	Average Percent Recovery of *R. solani* from Root Aamples^x^	
	2016	2017
Arugula ‘Astro’ + Solarization	70.67 bc^y^	66.67 c
Mustard green ‘Amara’ + Solarization	63.33 c	67.33 c
Turnip ‘Purple top forage’ + Solarization	78.00 abc	66.00 c
Solarization alone	81.25 abc	72.50 bc
Compost	86.67 ab	91.33 ab
Mustard meal	77.33 abc	75.33 abc
Non-treated control	92.00 a	93.33 a
*P*-value	0.0001	<.0001

^x^ For each plant, ten randomly selected flowering cherry root samples were plated on *Rhizoctonia* semi-selective medium to determine the percent recovery of *R. solani* from root samples. ^y^ Values are the means of three replicates; treatments followed by the same letter within a column are not significantly different at *P* ≤ 0.05.

**Table 4:** Cover crops and organic inputs used in this study.

Treatment	Scientific Name	Company	Rate
Arugula ‘Astro’	*Eruca vesicaria* (L.) Cav. ssp. *sativa* (Mill.) Thell.	Johnny’s Selected seeds Winslow, ME	8.3 × 10^5^ seeds/A
Mustard green ‘Amara’	*Brassica carinata* A. Braun	Johnny’s Selected seeds	2.3 × 10^6^ seeds/A
Turnip ‘Purple top forage’	*B. rapa* L.	Johnny’s Selected seeds	1.8 × 10^6^ seeds/A
Compost cow manure		Farm Fuel Inc. Freedom, GA	50 tons/A
Mustard meal		Farmers Organic Newton, GA	968 lb/A
